# Model-based optimal PEEP in mechanically ventilated ARDS patients in the Intensive Care Unit

**DOI:** 10.1186/1475-925X-10-64

**Published:** 2011-07-27

**Authors:** Ashwath Sundaresan, J Geoffrey Chase, Geoffrey M Shaw, Yeong Shiong Chiew, Thomas Desaive

**Affiliations:** 1 Department of Mechanical Engineering, College of Engineering, University of Canterbury, Private Bag 8140, Christchurch, New Zealand; 2 Department of Mechanical Engineering, University of Canterbury, Private Bag 8140, Christchurch, New Zealand; 3 Department of Intensive Care, Christchurch Hospital, Private Bag 4710, Christchurch, New Zealand; 4 Cardiovascular Research Center, Institute of Physics, Allée du 6 Août, 17 (Bât B5), B4000 Liège (Belgium

**Keywords:** Mechanical Ventilation, PEEP, Model Based Methods, ARDS

## Abstract

**Background:**

The optimal level of positive end-expiratory pressure (PEEP) is still widely debated in treating acute respiratory distress syndrome (ARDS) patients. Current methods of selecting PEEP only provide a range of values and do not provide unique patient-specific solutions. Model-based methods offer a novel way of using non-invasive pressure-volume (PV) measurements to estimate patient recruitability. This paper examines the clinical viability of such models in pilot clinical trials to assist therapy, optimise patient-specific PEEP, assess the disease state and response over time.

**Methods:**

Ten patients with acute lung injury or ARDS underwent incremental PEEP recruitment manoeuvres. PV data was measured at increments of 5 cmH_2_O and fitted to the recruitment model. Inspiratory and expiratory breath holds were performed to measure airway resistance and auto-PEEP. Three model-based metrics are used to optimise PEEP based on opening pressures, closing pressures and net recruitment. ARDS status was assessed by model parameters capturing recruitment and compliance.

**Results:**

Median model fitting error across all patients for inflation and deflation was 2.8% and 1.02% respectively with all patients experiencing auto-PEEP. In all three metrics' cases, model-based optimal PEEP was higher than clinically selected PEEP. Two patients underwent multiple recruitment manoeuvres over time and model metrics reflected and tracked the state or their ARDS.

**Conclusions:**

For ARDS patients, the model-based method presented in this paper provides a unique, non-invasive method to select optimal patient-specific PEEP. In addition, the model has the capability to assess disease state over time using these same models and methods.

## 1.0 Introduction

Patients suffering from acute respiratory failure, such as Acute Respiratory Distress Syndrome (ARDS), are often admitted to the Intensive Care Unit (ICU) and require Mechanical Ventilation (MV). ARDS mortality rates range from 30% to 70% [1]. In ARDS, the lung is inflamed and filled with fluid, becoming stiff, and lung units can collapse from the weight of additional fluid reducing the number of functional units. Thus, the ARDS lung is a stiffer, smaller lung, the so called "baby lung" [2]. There are no specific treatments for acute respiratory diseases, except to facilitate an environment for patients to recover [3].

Positive pressure ventilation has been highlighted as a key component of care [4,5], and is used to aid the recovery by reducing the work of breathing or taking over this work completely. Application of Positive End-Expiratory Pressure (PEEP) is one of the most important interventions in managing a patient with ARDS [6,7]. PEEP is applied to prevent de-recruitment at the end of expiration by keeping unstable lung units open and to recruit new lung units [7,8].

Each patient and their disease state are unique. Thus, MV management needs to be individualized. As the patient's condition changes, ventilator parameters need to be updated. Specifically, the level of PEEP needs to be adjusted to optimize recruitment and gas exchange, and to facilitate reductions in MV support, as patient condition improves.

Prior studies have been conducted to identify optimal ventilation management, including the use of low tidal volumes [6,9-11], and selecting PEEP using the inflection points of the pressure-volume (PV) curve. Although the use of low tidal volume is now common practice [12], the optimal PEEP is still debated. Selecting PEEP between the lower and upper inflection points provides a guide, but still does not provide a unique, patient-specific value.

Model-based recruitment tools provide a new method of determining optimal PEEP [13]. Sundaresan et al developed a minimal model that estimated recruitability of individual patients by fitting lung mechanics to measured PV curves. Although the research developed and validated the model with retrospective clinical data, its clinical feasibility was not illustrated. This paper validates this model in pilot clinical trials. The paper examines the model's ability to assess lung status, optimise PEEP and monitor patient condition over time.

## 2.0 Recruitment Model and Decision Support

### 2.1 Model Basics

**Figure 1:**
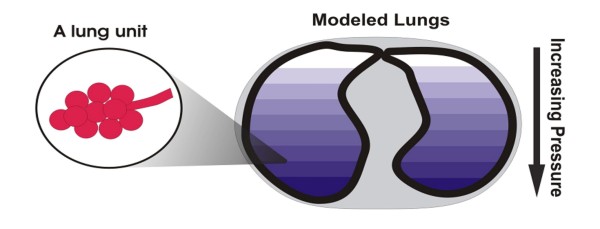
**Lung is modelled as a collection of units, evenly divided into compartment of different superimposed pressure**. Units comprising several alveoli and respiratory airway are located within each level. A unit may be either recruited or un-recruited in state (open or closed, in effect).

The lung mechanics model of Sundaresan et al [13] considers the lung as a collection of multiple lung units. Units represent a set of distal airways and alveoli, with the lung divided into several "horizontal" compartments. The model in Figure 1 works on the principle that units can only be either recruited or de-recruited.

Recruitment and de-recruitment of the modelled lung units are controlled by the distribution of Threshold Opening Pressure (TOP) and Threshold Closing Pressure (TCP). TOP is the critical pressure at which a previously collapsed unit is recruited during inspiration. TCP is the critical pressure where a previously recruited unit collapses during expiration. The model assumes that TOP and TCP are normally distributed [13,14]. Thus, these distributions are described by two variables: standard deviation (SD) and mean. The shapes of the distributions are unique to the patient's condition and the state of their disease, and will thus evolve with patient condition.

Given data from clinical PV loops, the model evaluates TOP and TCP distributions for each limb of the breathing cycle at a given PEEP. In particular, the model evaluates the TOP and TCP mean and SD for each PEEP level. As PEEP changes, the mean TOP and TCP also shift to reflect changes in recruitment, while SD remains constant. Thus, the model takes readily available PV measurements and outputs a TOP and TCP distribution for a given level of PEEP. This model has been validated using retrospective data [13].

### 2.2 Decision Support Metrics

#### TOP & TCP

To optimise PEEP, the clinician must have the ability to assess how recruitable a patient is with respect to PEEP. The slope of the TOP mean shift yields information on the patient-specific level of recruitability. Similarly, TCP gives information on alveolar retention.

**Figure 2:**
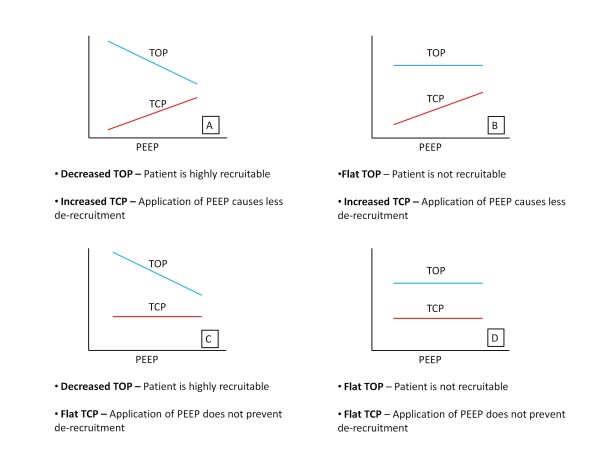
**Combinations of TOP and TCP mean shift**.

A large decrease in mean TOP with added PEEP implies that additional PEEP produces additional recruitment of new alveoli units. Similarly, If TCP increases, then the application of PEEP continues to prevent de-recruitment of unstable units and added PEEP is beneficial. Thus, PEEP might be selected on the basis of how TOP and TCP change with PEEP. Figure 2 shows the possible combinations of TOP and TCP shifts as a function of PEEP.

The application of PEEP can risk inducing ventilation-induced lung injury (VILI) [15,16]. Thus, when the mean TCP is greater than PEEP, more than 50% of alveoli will de-recruit during expiration. Hence, where PEEP and TCP mean are equal represents the PEEP at which no more than 50% of alveoli de-recruit, which is used here as a benchmark for setting PEEP.

#### Net Recruitment

**Figure 3:**
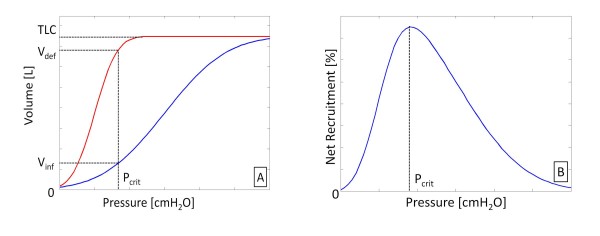
**Static PV curve and net recruitment.** (A) Static PV curve during inflation and deflation. Hysteresis is shown, with the volume at inflation, Vinf, much lower than volume during deflation, Vdef, for a given pressure, Pcrit. (B) Net recruitment as a function of pressure. Pcrit indicates pressure where net recruitment is maximised. At pressures below Pcrit, the rate of de-recruitment increases, while pressures above Pcrit, the rate of recruitment decreases.

Figure 3(A) shows a static PV curve for a lung with total lung capacity (TLC). If a PEEP equal to P_crit _is applied, the volume during inflation is represented by the volume V_inf_. However, during the deflationary portion of the static curve, the lung experiences a level of hysteresis and results in much higher lung volume for a given pressure, V_def_. The percentage of lung recruited at a given pressure is the lung volume divided by the TLC. Thus, during inflation for a given pressure, the percentage recruited volume is V_inf _divided by the TLC. Simultaneously, during deflation, the percentage alveoli remaining recruited at expiration is the V_def _divided by TLC. The difference between the percentage of alveoli remaining recruited at expiration and percentage recruited during inflation if PEEP equals P_crit _is the net level of recruitment and varies as a function of pressure.

Though the mechanism of hysteresis is still not fully understood, studies have shown that the larger the hysteresis, the higher the recruitability of the lung [17]. This result implies airway pressures should be increased to provide additional recruitment to the point where net recruitment does not rise with PEEP (Figure 3(B)). Note that net recruitment should only be used if the TOP continues to decrease with PEEP.

### 2.3 Disease State Metrics

Four other parameters are used to assess how ARDS disease state evolves over time.

#### Mean-Time Metric

**Figure 4:**
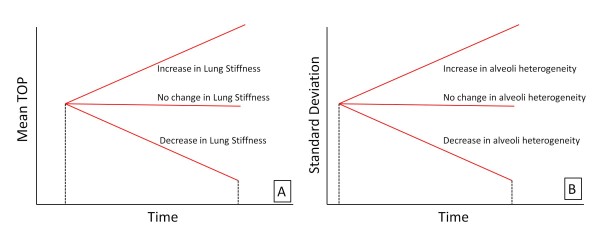
**TOP and model standard deviation as a function of time.** (A) Change in TOP as a function of time. Metric provides information with a change in lung condition and stiffness, and also provides information to the overall disease state. (B) Model standard deviation as a function of time.

As a patient's condition changes, the magnitude of the mean TOP for a given PEEP also changes. Increasing mean TOP indicates increasing lung stiffness and difficulty recruiting. A decrease implies more lung units available at a given pressure, and the lung has become less stiff. The trend of TOP can take on three possible scenarios over time, as shown in Figure 4(A).

#### Compliance-Time Metric

The second metric uses the SD to assess how the compliance of the lung changes with time, as shown in Figure 4(B). Schiller et al [18] showed that alveoli with various levels of injury typically appear in the same region of the lung, and even in the same microscopic field, because the ARDS lung is highly heterogeneous [19,20]. However, injured units recruit at higher pressures, while healthier units recruit at lower pressures. Therefore, changes in SD can help capture how healthy and damaged units are distributed as their relative percentages and compliance change. An increase over time in SD could be interpreted as more injured lung units being present in the lung. Similarly, a decrease in SD could be attributed to a reduction and corresponding improvement in disease state.

#### Top Gradient Metric

The gradient of the TOP mean shift in Figure 2 captures changes in the patient's recruitment response to PEEP. Changes in this gradient show how the recruitability response varies. An increase in magnitude of the TOP gradient implies the patient is becoming more responsive to PEEP, and a decrease implies less response.

#### Tcp Gradient Metric

The gradient of the TCP mean shift similarly shows how PEEP affects de-recruitment. An increase suggests that fewer alveoli are de-recruiting as a function of PEEP, and a decrease implies the application of PEEP does not prevent de-recruitment.

Importantly, healthy patients have all available alveoli recruited. Thus, these healthy patients will exhibit flat TOP and TCP gradients. However, this analysis only considers MV of very ill patients where the TOP and TCP gradient can vary significantly.

## 3.0 Methods and Materials

### 3.1 Patients

Ten patients were enrolled in this study from February 2010 through to September 2010. Of the ten patients enrolled, two patients underwent multiple recruitment manoeuvres over several days. The study was approved by the Upper South Island Regional Ethics Committee, New Zealand and was conducted in the Department of Intensive Care, Christchurch Hospital, New Zealand.

Patients were enrolled if they were over the age of 16 and were on MV therapy. Patients were only included if they were diagnosed with acute lung injury: a ratio of the partial pressure of arterial oxygen to the fraction of inspired oxygen (PF) of less than 300, but greater than 150. Patients were excluded if they were likely to be discontinued from MV therapy in 24 hours, were moribund or not expected to survive for greater than 72 hours, were diagnosed with asthma, had significant brain injury or required sedation.

### 3.2 Measurements

All patients were ventilated using volume controlled ventilation with the tidal volume selected by the clinician as 6 ml/kg and not changed for the duration of the trial. All patients underwent a protocolised recruitment manoeuvre with airway pressure and volume data collected. Heart rate, blood pressure and body temperature were also recorded. Before the recruitment manoeuvre and 30 minutes post manoeuvre, an arterial blood gas was taken to measure the PF ratio. Consented patients were given muscle relaxants to prevent spontaneous breathing efforts.

Patients were ventilated using a Puritan Bennett PB840 ventilator (Covidien, Boulder, CO, USA) in the Department of Intensive Care, Christchurch Hospital, New Zealand. A Hamilton Medical flow sensor (Hamilton Medical, Switzerland) was attached to the y-piece of the tubing and connected to a calibrated pneumotachometer. The pneumotachometer was used to obtain the pressure and flow measurements, and could capture the volume changes due to PEEP. A standard Dell™ (Dell, Austin, TX, USA) laptop was used in conjunction with Labview Signal Express (National Instruments, Austin, TX, USA) to obtain the raw measurements.

### 3.3 Recruitment Manoeuvre

The protocol used in this study was based on the work by Gattinoni et al [21]. The protocol involved a PEEP trial, with PEEP incremented in steps of 5 cmH_2_O and peak airway pressure limited to 45 cmH_2_O. Once all the required equipment was connected to a patient, PEEP was reduced to zero (ZEEP). Five PV curves were obtained under ZEEP conditions. During ZEEP, at the end of expiration, a volume hold is performed to measure inspiratory resistance. In addition, an inspiration hold is also performed to measure auto-PEEP. PEEP is then incremented in steps of 5 cmH_2_O and the corresponding PV curves obtained. PEEP is continually increased to PEEP_max _until peak airway pressure is equal to 45 cmH_2_O. Once PEEP max is achieved, PEEP is decremented in steps of 5 cmH_2_O back to the initial ventilation settings. In all patients, tidal volume was held constant at 500 ml or 6 ml/kg, whichever was lower.

## 4.0 Results

### 4.1 Peep Selection

**Figure 5:**
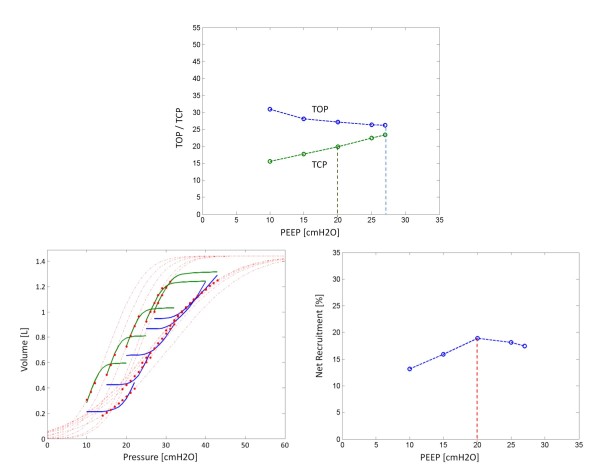
**Patient 1: TOP and TCP as a function of PEEP**. Bottom left is the model fit. Bottom right is net recruitment over PEEP.

All data showed the expected linear increase in TCP, and a decrease in TOP as a function of PEEP. Table 1 summarises the best fit threshold pressure distribution parameters and the average fitting errors for Patient 1 and excludes PV curves with PEEP levels below the auto-PEEP of 10 cmH_2_O. Figure 5 illustrates the TOP and TCP curves for Patient 1, along with the model fit and net recruitment curves.

**Table 1:** Model fitting error for Patient 1

PATIENT 1				Number of Units		144000
				Inflation SDM		15
				Deflation SD		7
				Auto-PEEP [cmH_2_O]		10
	
		Inflation			Deflation	
**PEEP [cmH_2_O]**	**Mean[l/cmH_2_O]**	**Error [ml]**	**Error [%]**	**Mean[l/cmH_2_O]**	**Error [ml]**	**Error [%]**

10	30.97	22.17	7.86	15.57	15.48	4.61
15	28.07	22.50	4.44	17.68	5.61	1.02
20	27.12	22.27	3.11	19.88	5.72	0.63
25	26.41	21.71	2.05	22.43	7.47	0.68
27	26.18	15.11	1.34	23.39	2.73	0.24

The model fits all the clinical data well as shown by low percentage error in Table 2. Fitting errors for all patients are summarised in Table 2. In all cases, patient-specific standard deviation was held constant for each patient across all PEEP values in a given trial. The fitting errors are presented as average absolute volume fitting errors and as percentage errors. The optimal patient-specific PEEP depending on the TOP, TCP and net recruitment are summarised in Table 3, including the auto-PEEP identified, for all patient trials.

**Table 2:** Summary of absolute fitting errors for all patients

	Inflation		Deflation	
	
	Error [ml]	Error [%]	Error [ml]	Error [%]
Median	19.47	2.50	6.96	0.82
Interquartile Range (IQR)	[12.36 - 22.79]	[1.56 - 4.62]	[4.51 - 13.22]	[0.54 - 2.34]
90% Confidence Interval	[7.83 - 49.95]	[0.83 - 19.21]	[2.12 - 33.35]	[0.19 - 10.75]

**Table 3:** Summary of auto-PEEP and model-based PEEP selection metrics for all patients

	**Auto-PEEP [cmH**_**2**_**O]**	**Clinically Selected PEEP [cmH**_**2**_**O]**	Inflation SD	**Model-Based PEEP Selection [cmH**_**2**_**O]**		
				
				TOP	TCP	Net Recruitment
Patient 1	10	10	15	27	20	20
Patient 2	2	12	11	15	15	15
Patient 3	0	10	12	10	15	20
Patient 4	9	10	25	20	20	30

Patient 5 - Trial 1	13	12	16	20	25	25
Patient 5 - Trial 2	8	12	15	20	25	20

Patient 6 - Trial 1	10	11	11	15	20	20
Patient 6 - Trial 2	3	13	14	15	15	20
Patient 6 - Trial 3	2	10	14	10	20	15

Patient 7	2	7.5	10	5	10	10
Patient 8	0	12	15	15	20	30
Patient 9	12	10	15	25	20	29
Patient 10	3	10	16	15	20	15

### 4.2 Monitoring Disease Evolution

**Figure 6:**
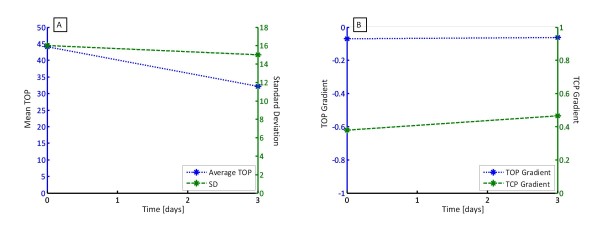
**(A) Average TOP and SD over time for Patient 5 and (B) TOP and TCP gradient over time**.

**Figure 7:**
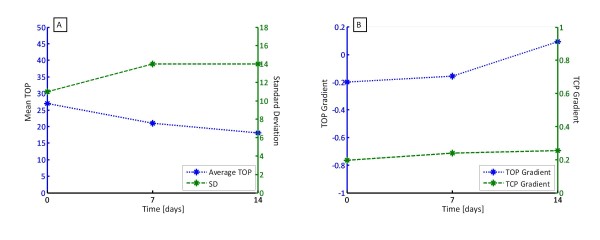
**(A) Average TOP and SD over time for Patient 6 and (B) TOP and TCP gradient over time**.

Patients 5 and 6 had multiple trials and recruitment manoeuvres on different days. The purpose was to examine the ability to track the evolution of disease state with time. Patient 5 had two trials, with the second performed three days after the first. Patient 6 had three trials, with the subsequent two manoeuvres performed 7 and 14 days later. The variations of TOP, SD, TOP gradient and TCP gradient are shown in Figure 6 for Patient 5 and Figure 7 for Patient 6, with summary data in Table 3.

Figure 6(A) shows the average TOP and SD over time for Patient 5, and shows that a significant drop in average TOP, and a negligible drop in SD. Hence, Patient 5 has improved slightly in the ability to recruit and is slightly more compliant. Figure 6(B) shows no change in TOP gradient and little change in TCP gradient, suggesting that the recruitment response to PEEP has not changed significantly.

Similar to Patient 5, Patient 6 exhibits a decrease in average TOP, indicating recruitment has improved with time over the three trials. However, Figure 7(A) also indicates that between the first and second trial, SD has increased, implying less compliance and an increase in ARDS affected alveoli. Prior to the trial, the clinician hypothesised that this patient had severe ARDS. The increased level of recruitment could be attributed to marginally unhealthy units being recruited after a sustained level of pressure. However, the increase in SD suggests that the condition of unhealthy alveoli was getting worse or that more alveoli were becoming ARDS affected. Patient 6 died later of severe respiratory failure, supporting this hypothesis. Figure 7(B) supports these outcomes, suggesting that the patient was less responsive to PEEP induced recruitment over time, particularly between the second and third trials.

## 5.0 Discussion

### 5.1 Peep Selection

Based on the results of these clinical trials, the model highlighted aspects of MV that are clinically important in determining optimal PEEP. First, the model evaluates waveforms to assess recruitment and de-recruitment, thus determining the patient's recruitability. By examining how recruitable a patient is with respect to TOP, TCP, and net rate of recruitment, the model non-invasively evaluates the point where additional PEEP does not cause additional recruitment or retention of alveoli.

The model uses the TOP to assess the impact on recruitability using PEEP. The clinical data suggested that Patients 1, 3, 4, 9 and 10 were highly recruitable. These patients showed a significant drop in TOP as PEEP increased. In contrast, Patients 2, 6, 7 and 8 showed negligible or no change in TOP as PEEP increased, with PEEP resulting in minimally additional or no recruitment. Patient 5 underwent two trials, and in trial 1, was not very recruitable due to the minimal changes seen in TOP. However, during the second trial, Patient 5 had a stronger recruitment response to PEEP.

The model also uses TCP to evaluate optimal PEEP, where the aim is to prevent de-recruitment or maintain recruited alveoli. All ten patients showed an increase in TCP as PEEP increased. This suggests that continual increases in PEEP will at least minimise de-recruitment, and PEEP should be maximised. However, because high PEEP can result in VILI [15,16], the PEEP where no more than 50% of alveoli de-recruit was chosen as the optimal PEEP in this analysis. Although this 50% level is arbitrary, it represents a trade off between patient safety and ventilation efficacy.

**Figure 8:**
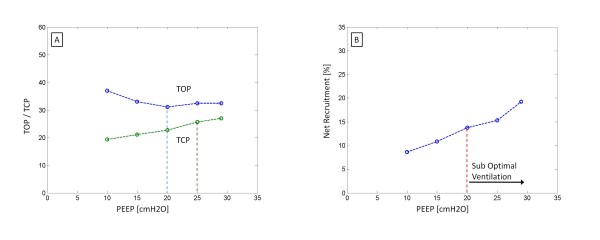
(A) TOP and TCP vs PEEP for Patient 5, Trial 2 and (B)  Net recruitment for Patient 5, Trial 2 indicating sub-optimal ventilation beyond 20 cmH2O.

Finally, the model also used net recruitment to select PEEP. This metric is only valid when the TOP decreases. Patients 2, 5 (trial 2), 6 (trials 1 and 3) and 7 showed an increase in TOP at high levels of PEEP, as shown in Figure 8(A). Based on alveolar recruitability, an increase in TOP would imply that high PEEP results in volume lost. Physiologically, this outcome would imply that there are more alveoli being damaged or lost (due to over inflation) than recruited. However, this sudden change of patient condition is highly unlikely due to the relatively low pressures used in this trial.

The clinical data showed this increase in TOP occurred when the compliance of the dynamic PV curve decreased markedly. When the compliance of the lung decreases, the pressure required to deliver a given volume of air increases. This result can occur when alveoli are maximally recruited and begin to over-inflate [16]. Thus, an increase in PEEP results in alveolar over-inflation, rather than additional recruitment, and ventilating at higher PEEP is similar to ventilating above the upper inflection point (UIP). Therefore, the increase in TOP is an indication of alveolar over-inflation thus justifying the conclusion that it results in sub-optimal ventilation, as can be seen in Figure 8(B) for Patient 5.

### 5.2 Compliance Changes

The model used a constant SD across PEEP within a given trial to reflect the disease state of a patient, as fitted to the largely linear portion of the dynamic PV curve. When the model was applied to the clinical data, it was evident that the standard deviation is not necessarily constant across all PEEP values considered in this study. At relatively low and high PEEP, the compliance can be significantly different than in the linear portion of the static PV curve [22]. The variation in compliance is shown in Table 4 for the patients in this study. The compliance values are unusually high compared to the compliance for ARDS patient as in previous work [6,21,23-25]. This could be attributed to the presence of auto-PEEP in patients. Regardless of this limitation, it still reflects the recruitment response to PEEP indicating the robustness of the model.

**Table 4:** Variation in compliance across all patients

	**PEEP in linear region [cmH**_**2**_**O]**	**Compliance in Linear region [ml/cmH**_**2**_**O]**	**Highest PEEP [cmH**_**2**_**O]**	**Compliance at highest PEEP [ml/cmH**_**2**_**O]**
Patient 1	15 - 25	[45.43 - 60.47]	27	34.92
Patient 2	5 - 20	[63.19 - 103.91]	22	58.34
Patient 3	5 - 20	[70.62 - 107.96]	28	25.70
Patient 4	10 - 20	[21.82 - 44.81]	30	48.45
Patient 5 - Trial 1	15 - 20	[35.75 - 55.57]	25	24.40
Patient 5 - Trial 2	10 - 20	[55.01 - 62.21]	29	26.94
Patient 6 - Trial 1	10 - 20	[30.24 - 58.69]	25	17.02
Patient 6 - Trial 2	10 - 20	[35.13 - 53.80]	25	33.70
Patient 6 - Trial 3	5 - 15	[48.01 - 77.45]	20	23.64
Patient 7	5 - 10	[12.39 - 23.48]	16	5.76
Patient 8	10 - 25	[40.16 - 60.43]	30	32.14
Patient 9	10 - 25	[50.30 - 82.93]	30	36.70
Patient 10	10 - 25	[30.38 - 44.68]	30	26.74

The preliminary validation performed in Sundaresan et al [13] used retrospective clinical data with PV curves obtained during 30 minutes of sustained pressure [26] and showed similar compliance across the different PEEP. Recruitment manoeuvres applying sustained pressure for relatively long periods of time have been shown to improve recruitability [27]. This approach may have resulted in the very constant compliance in the data used in the initial validation on this data set. The model required multiple PV loops to assess the recruitability of the patient. From a practical perspective, if multiple PV loops are required, it is not feasible to hold the PEEP for 30 minutes as part of a clinical protocol for everyday use. Thus, in this situation, a short recruitment manoeuvre would be more appropriate clinically, as used in these clinical trials.

**Figure 9:**
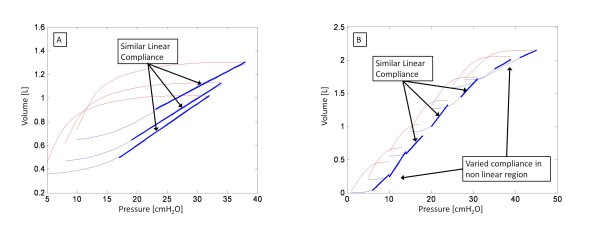
**PV curves comparison.** (A) PV curves for a dataset from  Bersten et al. showing similar linear compliance. (B) PV curves from clinical data. Similar compliance is exhibited in the linear portion, but compliance significantly varies at low and high PEEP.

Figure 9(A) shows PV curves from the Bersten dataset and Figure 9(B) from this study. Figure 9(A) shows the linear compliance not varying significantly in the three curves. In contrast, Figure 9(B) shows compliance varying at extreme PEEP, but remaining relatively constant in the linear region. Thus, although 30 minutes of sustained PEEP is not performed for the clinical dataset, the relatively constant compliance within the linear region is still obtained, which still incorporates the clinically acceptable PEEP ranges. In addition, the data from Bertsen used a maximum PEEP of 15 cmH_2_O, and did not push PEEP to the higher values compared to the trials used in this study. Thus, the relatively constant compliance seen by Bersten could have also been attributed to the fact that PEEP was not tested at extreme values.

### 5.3 SD & Compliance

**Figure 10:**
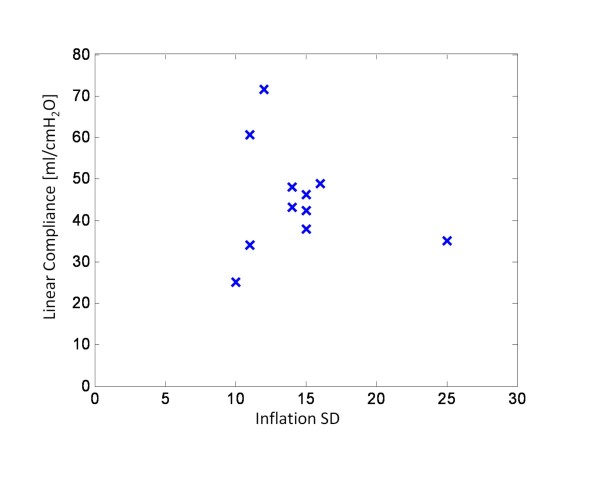
**SD vs linear compliance for all patients**.

Compliance in the linear portion of the static PV curve has been reported to be between 20 and 61 ml/cmH_2_O for patients with ALI and ARDS [6,21,23-25]. Table 5 summarises the inflation SD and the corresponding compliance of the linear portion of the static PV curve, with the results plotted in Figure 10. The aim is to examine the strength of the correlation between SD and compliance.

**Table 5:** SD and linear compliance for each clinical patient

	Inflation SD	**Linear Compliance [ml/cmH**_**2**_**O]**	TLC [ml]	**Normalised Linear Compliance [ml/cmH**_**2**_**O]**	TLC [ml]
Patient 1	15	37.79	1440	26.25	1000
Patient 2	11	60.52	1710	35.40	1000
Patient 3	12	71.66	2200	32.48	1000
Patient 4	25	34.94	2200	15.88	1000
Patient 5 - Trial 1	16	48.80	1980	24.61	1000
Patient 5 - Trial 2	15	42.26	1610	26.25	1000
Patient 6 - Trial 1	11	33.92	960	35.36	1000
Patient 6 - Trial 2	14	47.97	1710	28.02	1000
Patient 6 - Trial 3	14	43.21	1540	28.04	1000
Patient 7	10	25.11	650	38.57	1000
Patient 8	15	46.20	1760	26.23	1000
Patient 9	15	49.58	1890	26.23	1000
Patient 10	16	36.17	1470	24.60	1000

Compliance was normalised to allow correction for varying TLC.

**Figure 11:**
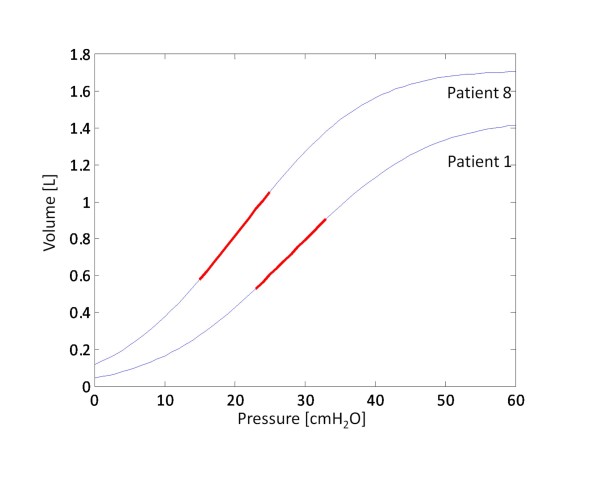
**Static PV curve for Patient 1 and Patient 8**. Red solid lines show the linear compliance for both patients. SD is identical for both patients, but TLC is different, causing linear compliance to vary.

**Figure 12:**
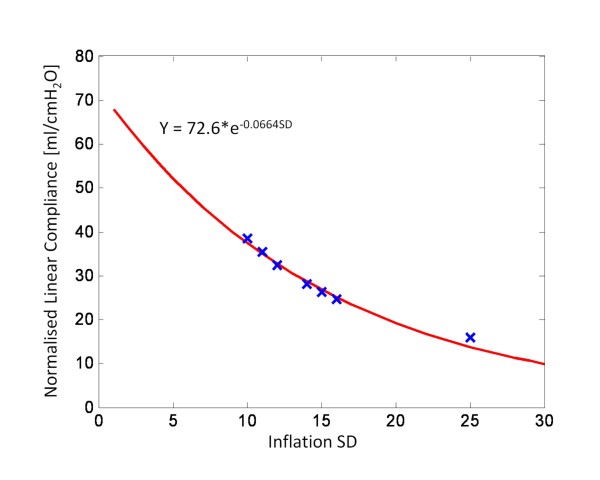
**SD vs normalised linear compliance for all patients**.

Figure 10 suggests that there is no strong correlation between inflation SD and the linear compliance. However, this result could be attributed to the fact that the TLC varies significantly between patients. For example, Patient 1 and Patient 8 exhibit the same SD, but the linear compliance is different, as shown in Figure 11 due to differences in TLC. To correct for the varying lung capacity, the compliance was normalised, such that all patients exhibit a TLC of 1 L. This normalization effectively scales the static PV curve, and thus, modifies the compliance. The normalised compliance values thus present a fair comparison and are shown in Table 5, and plotted in Figure 12.

Figure 12 shows the normalised compliance as a function of SD and a very strong exponential relationship. In essence, the SD captures the effect of compliance. Although, numerically, SD may not accurately represent true linear compliance, these results show that SD can be effectively used as an indicator of the diseased state across a specific patient and across many patients if normalised.

### 5.4 Effect of Inspiratory Resistance

**Figure 13:**
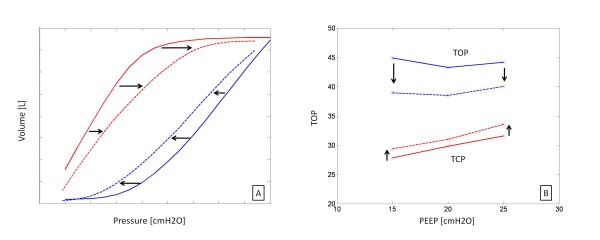
**Correction for resistive pressure. **(A) PV curve takes  on a narrower shape when corrected for resistive pressure. (B) TOP and TCP magnitude change when model is fitted to PV loops with resistive pressure removed for Patient 5, Trial 1.

The effect of endotracheal tube resistance was evaluated by performing an inspiratory hold during the deflation to ZEEP and measuring plateau pressure. The difference in peak pressure and plateau pressure was attributed to the pressure loss of the tube. Because a known decelerating flow waveform was used, inspiratory resistance can be calculated. Correcting for resistive pressure, the PV curves takes on a narrower shape, as shown in Figure 13(A).

The model was re-fitted with the corrected PV curves, and the resulting TOP and TCP calculated, in Figure 13(B). As shown, the overall magnitude of the TOP drops, while the TCP increases. This result is expected, as the pressure to overcome resistance has now been partly compensated for. However, the overall TOP and TCP trends are still similar, and thus, the results, which are based on trends, are unchanged.

The inspiratory hold is an additional step when obtaining the PV curves. Although the magnitude of TOP and TCP changes when corrected for resistive pressure, the trend is similar, and still produces the same patient-specific PEEP response. Thus, from a practical perspective, not measuring the flow resistive component yields similar responses, and avoiding this step reduces clinical burden in obtaining PV loops.

### 5.5 Airway Obstructions

One of the major limitations of this model is the reliance on the quality of the dynamic PV curve. In particular, the model does not work when patients exhibit severe airway obstructions, as was the case with Patient 4. Patient 4 exhibited very high auto-PEEP of 9 cmH_2_O and, as a result, the model was only fitted to PV curves with PEEP above 10 cmH_2_O.

**Figure 14:**
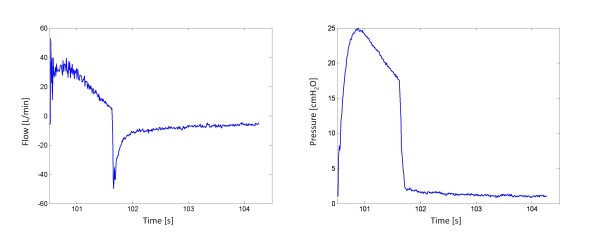
**Raw Flow vs Time and Pressure vs Time for Patient 4**.

Figure 14 shows the flow and pressure waveforms. The waveforms highlight an interesting scenario where airway pressure begins to decrease even though the ventilator is still delivering positive flow. More specifically, the volume of the lung continues to increase even though there is a pressure drop, and thus does so faster than incoming flow.

In patients with ARDS and ALI, the lung is very heterogeneous. Some areas of the lung are compliant and healthy, while other portions of the lung can be extremely stiff [28]. In addition to a heterogeneous distribution of alveoli, an ARDS lung can exhibit significant airway resistance due to the presence of fluid secretions within the airway. The drop in pressure may then be explained when one considers heterogeneity of the ARDS lung.

**Figure 15:**
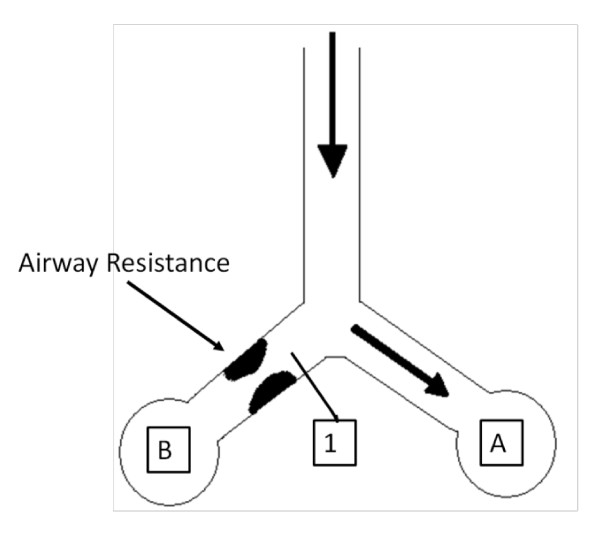
**Schematic highlighting the decreasing pressure phenomena with a pressure build up at Point 1**.

As more air flows into lungs, the airflow takes the path of least resistance first fills compliant, healthy alveoli, A, as shown in Figure 15. However, the volume of alveoli A is limited. Hence, pressure builds up in the airways, and, as pressure builds at point 1, flow eventually overcomes the resistance to recruit alveoli B, recruiting more volume.

In this trial, the flow waveform used was a decelerating flow pattern. When the pressure difference at point 1 overcomes the resistance, flow begins to enter alveoli B. However, because the flow is decelerating, the newly available volume is not filled fast enough by the decelerating airflow. Thus, although lung volume is still increasing, the pressure drops due to the relative lack of flow.

This pressure drop could also be indicative of the severity of the disease state. Patient 4 was the only one who exhibited this drop in pressure, implying severe airway restrictions similar to what a COPD patient experiences. The pressure drop indicates that there are severe resistances to airflow and highlight potentially extreme heterogeneity in the ARDS lung. Patient 4 later died due to advanced respiratory failure, further highlighting this possible indicator of the potential severity of ARDS.

Although Patient 4 showed such a pressure drop, the true compliance for this patient is when the pressure and volume increase (ie - when alveoli A is filling). Hence, the model is fitted to the regions of PV loops where pressure and volume are increasing. However, the problem with fitting to this region is that there are not many data points available. Hence, although the model can still fit to the PV curves, it may not give an accurate representation of patient recruitability, which is also a potential limitation.

## 6.0 Conclusions

The paper highlights the clinical validation and viability of using a lung mechanics recruitment model to assist in therapy. This paper introduced three metrics to help select optimal, patient-specific PEEP. Although the TOP, TCP and net recruitment metrics provide three different methods to evaluate the PEEP, they also provide a set of values that can be used in the clinic to gain insight into the patient's recruitment and de-recruitment with the application of PEEP. Comparisons to clinical settings show that optimal PEEP can be higher than what is currently set.

This research also introduced four additional metrics to assess how the disease state evolves with time. Tracking the TOP with time yields information on the recruitability of the patient, and how this changes with time. In addition, tracking the SD provides information on how compliant the patient is and the state of the disease with time. Finally, tracking the gradient of TOP or TCP with time provides information on how the patient's response to PEEP has changed. This metric strongly correlated with the clinical outcome of the two patients, and highlights the utility of using disease evolution metrics in the clinic.

For patients with ARDS, the model presented suggests that optimal non-invasive PEEP titration can be achieved. In particular, the different metrics developed give a different insight into the various mechanisms involved when selecting PEEP. The model outputs showed that optimal PEEP was much higher than clinically selected PEEP, and that most patients experienced auto-PEEP, indicating that current methods of ventilation still do not adequately ventilate patients.

## 7.0 Competing interests

The authors declare that they have no competing interests.

## 8.0 Authors contributions

AS, JGC and GMS created and defined the model. YSC and TD had input to analysis of results. GMS implemented trials clinically with input from all others. All authors had input in writing and revising the manuscript.
